# Establishment of a New Quality Control and Vaccine Safety Test for Influenza Vaccines and Adjuvants Using Gene Expression Profiling

**DOI:** 10.1371/journal.pone.0124392

**Published:** 2015-04-24

**Authors:** Haruka Momose, Takuo Mizukami, Madoka Kuramitsu, Kazuya Takizawa, Atsuko Masumi, Kumiko Araki, Keiko Furuhata, Kazunari Yamaguchi, Isao Hamaguchi

**Affiliations:** Department of Safety Research on Blood and Biological Products, National Institute of Infectious Diseases, Musashimurayama, Tokyo, Japan; Instituto Butantan, BRAZIL

## Abstract

We have previously identified 17 biomarker genes which were upregulated by whole virion influenza vaccines, and reported that gene expression profiles of these biomarker genes had a good correlation with conventional animal safety tests checking body weight and leukocyte counts. In this study, we have shown that conventional animal tests showed varied and no dose-dependent results in serially diluted bulk materials of influenza HA vaccines. In contrast, dose dependency was clearly shown in the expression profiles of biomarker genes, demonstrating higher sensitivity of gene expression analysis than the current animal safety tests of influenza vaccines. The introduction of branched DNA based-concurrent expression analysis could simplify the complexity of multiple gene expression approach, and could shorten the test period from 7 days to 3 days. Furthermore, upregulation of 10 genes, *Zbp1*, *Mx2*, *Irf7*, *Lgals9*, *Ifi47*, *Tapbp*, *Timp1*, *Trafd1*, *Psmb9*, and *Tap2*, was seen upon virosomal-adjuvanted vaccine treatment, indicating that these biomarkers could be useful for the safety control of virosomal-adjuvanted vaccines. In summary, profiling biomarker gene expression could be a useful, rapid, and highly sensitive method of animal safety testing compared with conventional methods, and could be used to evaluate the safety of various types of influenza vaccines, including adjuvanted vaccine.

## Introduction

Influenza is a globally important acute respiratory disease caused by a highly contagious virus, which leads to high fever, muscle aches, sore throat, cough, and sometimes death [1–3]. Preventative vaccination before endemic seasons can be helpful to control influenza, so influenza vaccines are manufactured annually to match predicted circulating strains. Influenza vaccines are usually produced in embryonated chicken eggs, and in the past, egg-derived vaccines have contained measurable amounts of egg proteins [4]. Substantial contamination by egg-derived components could cause adverse events in recipients with egg allergies [5]. The introduction of density gradient centrifugation and continuous-flow zonal ultracentrifuges has enabled to prepare highly purified influenza vaccines which have been delivered to many people [6, 7]. Despite the split influenza vaccines have successfully reduced the adverse events, egg-dependent platform still have some problems including the availability of large numbers of fertilized eggs. Recently, influenza vaccines of the next generation have been licensed for human use, such as adjuvant containing influenza vaccines and cell-derived influenza vaccines [8–11]. Adjuvant(s) containing influenza vaccines may save the amount of antigens, and may potentiate the immunogenicity of vaccines. Virosomes and oil-in-water emulsions are now commercialized in combination with influenza vaccines [12, 13]. Cell-derived influenza vaccines may overcome another problem of egg-derived vaccines when seed virus is difficult to grow in eggs, and a sufficient quantity of antigens required for vaccine production is difficult to prepare. A MDCK-derived influenza vaccine was first licensed by European Medicines Agency in 2007 and by the United States Food and Drug Administration in 2012 [14], and a Vero-derived influenza vaccine was licensed by the Austrian Agency for Health and Food Safety in 2010 [15,16].

Influenza vaccines for public use are made according to good manufacturing practices, and must be rigorously evaluated to assure their safety and efficacy in the pre-clinical and clinical studies. Furthermore, manufacturers must submit samples of vaccines to national control laboratories, and must be checked the quality of each batch to meet the specification of vaccine at the market authorization, before vaccines being released to the market. In Japan, several animal tests are applied for the quality control of vaccines including the abnormal toxicity test (ATT). In the ATT, vaccinated guinea pigs are monitored for 7 days, and no statistically significant difference (p = 0.01) in weight loss should be observed between the treated animals and the parental group, for which the weight loss data for previously satisfactory batches of the same kind of vaccine are known [17, 18]. The mouse leukopenic toxicity test (LTT), and the test for toxicity to mouse weight gain are also applied for influenza vaccines [17]. Although these animal tests have been played an important role in the quality control of influenza vaccines, there are several points to be improved for influenza vaccines of the next generation; the sensitivity to detect the remaining toxicity of vaccines should be increased, and the clarification of molecular mechanisms underlying the development of vaccine toxicity is desired. Furthermore, as the highly purified split vaccines are available and the incidence of adverse reactions is getting lower, the role of animal tests should be changed. It is required to seize minute biological changes corresponding to the administration of vaccines using more reliable and robust parameters such as biomarkers. To define the biological responsiveness of vaccine-treated animals, we have previously used a DNA microarray analysis to investigate the expression profiles in the tissues of vaccinated rats [19–23]. The profiles showed significant changes in the lungs 1 day after the administration of the whole virion influenza vaccine [20]. A subset of 17 genes (Table 1) was selected to confirm and validate the results of the DNA microarray analysis with quantitative RT-PCR. *Mx2*, *Ifi47*, *Ifrd1*, *Trafd1* and *Irf7* are interferon-stimulated genes (ISGs). *Zbp1* plays a role in the induction of ISGs. *Psme1*, *Psmb9*, *C2*, *Tap2* and *Tapbp* are related to antigen modification and presentation. *Lgals3bp*, *Lgals9*, *Cxcl9*, *Cxcl11*, *Csf1* and *Timp1* are related to chemokine and cytokine signaling. These biomarkers were upregulated in the rat lung treated with the whole virion influenza vaccine treatment [20]. In our previous study, we also applied concurrent gene expression analysis by branched DNA (bDNA)-based method to the gene expression analysis of biomarkers [23]. In the bDNA-based method, targeted mRNAs are captured by color-coded magnetic beads. The targeted mRNAs are also sequentially hybridized with target-specific probes, bDNA-shaped amplifiers, and a biotinylated probe to build a signal amplification tree. The signal, which correlates with the expression level of the gene, is detected with the addition of streptavidin-phycoerythrin. The expression profiles of 17 genes were well correlated to the change in body weight and leukocyte counts upon whole virion vaccines treatment [23], and 17 genes would be useful biomarkers for influenza vaccines (Table 1).

**Table 1:** Biomarkers for influenza vaccines.

class	symbol	name	Accession number
Grade 1 (< 10%)	*Cxcl11*	Chemokine (C-X-C motif) ligand 11	NM_182952
	*Cxcl9*	Chemokine (C-X-C motif) ligand 9	NM_145672
	*Zbp1*	Z-DNA binding protein 1	NM_133564
	*Mx2*	Myxovirus (influenza virus) resistance 2	NM_134350
	*Irf7*	Interferon regulatory factor 7	NM_001033691
	*Lgals9*	Lectin, galactoside-binding, soluble, 9	NM_012977
Grade 2 (< 20%)	*Ifi47*	Interferon gamma inducible protein 47	NM_172019
	*Tapbp*	TAP binding protein (tapasin)	NM_033098
	*Csf1*	Colony stimulating factor (macrophage)	NM_023981
	*Timp1*	TIMP metallopeptidase inhibitor 1	NM_053819
	*Trafd1*	TRAF type zinc finger domain containing 1	NM_053760
	*Lgals3bp*	Lectin, galactoside-binding, soluble, 3 binding protein	NM_139096
	*Psmb9*	Proteasome (prosome, macropain) subunit, beta type, 9	NM_012708
Grade 3 (< 40%)	*C2*	Complement component 2	NM_172222
	*Tap2*	Transporter 2, ATP-binding cassette, sub-family B (MDR/TAP)	NM_032056
	*Ifrd1*	Interferon-related developmental regulator 1	NM_019242
	*Psme1*	Proteasome (prosome, macropain) activator subunit 1	NM_017264

In this study, we firstly investigated whether gene expression patterns correlate with, and show higher sensitivity than the conventional animal tests for detecting reactivity of a wide variety of influenza vaccines. Secondly, we applied our new gene expression analysis to the quality control of a cell-based vaccine and an adjuvanted-containing vaccine to investigate whether biological responsiveness of vaccinated animals was associated with expression profiles of our biomarkers.

## Materials and Methods

### Animals and Ethics statement

Eight week-old male Wistar rats weighing 160–200 g were obtained from SLC (Tokyo, Japan). Rats were housed in rooms maintained at 23 ± 1°C, with 50 ± 10% relative humidity, and 12 h light/dark cycles for at least one week prior to the test challenge. All animal experiments were performed according to the guidelines of the Institutional Animal Care and Use Committee of the National Institute of Infectious Diseases (NIID), Tokyo, Japan. The study was approved by the Institutional Animal Care and Use Committee of NIID.

### Vaccines

National reference of influenza vaccine (RE) is a toxicity reference issued by NIID (Japan). RE is a lyophilized whole virion preparation of inactivated influenza virus, used as a toxicity reference in a leukopenic toxicity test in Japan [17]. It was reconstituted in 12 ml of physiological saline (SA) for a final concentration of 1 U/ml. Serial dilutions with SA were performed to prepare 1 U/ml, 0.5 U/ml and 0.25 U/ml solutions. The bulk materials of the HA vaccine (HA-A to HA-D), obtained during the manufacturing process, were kindly provided from four manufactures in Japan: Denka Seiken Co., Ltd., Kitasato Daiichi Sankyo Vaccine Co., Ltd., The Chemo-Sero-Therapeutic Research Institute, and The Research Foundation of Microbial Diseases of Osaka University. Serial dilutions with SA were performed to prepare 4-fold (4x, approximately 120 μg HA/ml each strain), 2-fold (2x, approximately 60 μg HA/ml each strain) and 1-fold (1x, approximately 30 μg HA/ml each strain which is equivalent to final bulk) concentrated HA bulk materials. Influenza HA vaccines manufactured from fertilized eggs (HA), from Vero cells (HA-cell, Baxter International Inc.), and the virosomal-adjuvanted HA vaccine (HA-v, Crucell, Berna Biotech AG) were purchased for research purposes. Five ml of each vaccine, HA bulk material, or SA was injected into the rat peritoneum. The vaccines contained the following: HA: A/California/7/2009 (H1N1), A/Victoria/210/2009 (H3N2), and B/Brisbane/60/2008, containing 30 μg HA/ml each strain; HA-cell: A/California/7/2009 (H1N1), A/Perth/16/2009 (H3N2), and B/Brisbane/60/2008, containing 30 μg HA/ml each strain; HA-v: A/California/7/2009(H1N1)-like, A/Perth/16/2009(H3N2)-like, and B/Brisbane/60/2008-like, containing 30 μg HA/ml each strain.

### Abnormal toxicity test (ATT) and leukopenic toxicity test (LTT)

ATT and LTT were performed according to the Minimum Requirements for Biological Products [17], using rats as test animals. For ATT, rats were weighed before and one day after intraperitoneal injection of each vaccine, and the percent weight loss was calculated. For LTT, peripheral blood was collected from the same rat, immediately mixed with EDTA (4.4 mM), and the number of white blood cells (WBC) and platelets (PLT) were counted using an automatic hematocytometer, the Celltac MEK-6450 (Nihon Kohden, Tokyo, Japan).

### Extraction of total RNA and preparation of lung lysate

The rats were anesthetized with diethylether, and one lung lobe was obtained from each rat 1 day after the vaccine was injected. Each lobe was immediately frozen in liquid nitrogen, homogenized, and mixed with an ISOGEN reagent (an acid guanidinium thiocyanate-phenol solution, NIPPON GENE, Tokyo, Japan). Total RNA was purified in accordance with the manufacturer’s instructions, and cDNA synthesis was conducted with a First-strand cDNA Synthesis Kit (Life Science, Inc., St. Petersburg, FL, USA). For lung lysate, one lung lobe was immediately stored in RNAlater (Invitrogen), homogenized in 3 ml of Homogenizing Solution (Panomics/Affymetrix, Fremont, CA, USA) containing 166 μg/ml Proteinase K, and incubated at 65°C for 30 min.

### Concurrent gene expression analysis by a branched DNA (bDNA)-based method

Concurrent gene expression analysis was performed by bDNA-based method, using the QuantiGene Plex 2.0 Assay Kit (Panomics/Affymetrix, Fremont, CA, USA). Either total RNA solution (bDNA-RNA) or tissue lysate (bDNA-lysate) can be used as starting materials.

Forty μl of lung lysate (50-fold dilution) or 20 μl of total RNA (1 ng/μl) was mixed with 33.3 μl of Lysis Mixture, 2 μl of Blocking Reagent, 1 μl of Mag Capture Beads, and 2 μl of the target gene-specific probe set (all reagents were contained in QuantiGene Plex 2.0 Assay Kit). Proteinase K (100 μg/ml) was also added for lysate analysis. Each sample mixture was incubated at 54°C for 18 h. After the unbound samples were washed away, signals for the bound target mRNA were developed by sequential hybridization with bDNA pre-amplifier, amplifier, and biotin-conjugated label probes, at 50°C for 1 h each, according to the manufacturer’s instructions. Streptavidin-conjugated phycoerythrin was added to the wells and incubated at room temperature for 30 min. The luminescence of each well was measured using a Luminex 100 system (Luminex, Austin, TX, USA) or a Bio-Plex 200 system (BioRad, Hercules, CA, USA). The 17 targeted genes and β-actin mRNA were concurrently quantified, and the ratio of the target gene to β-actin was calculated. Values below the luminescence counts of water (for bDNA-RNA) or those of Homogenizing Solution (for bDNA-lysate) were deemed to be zero.

### Statistical analysis

A statistical analysis was performed in GraphPad Prism 5 (GraphPad Software, La Jolla, CA) to determine the differences between SA and serial dilutions of RE, and the differences between SA and serial dilutions of the bulk materials of the HA vaccine, using one-way analysis of variance followed by a Tukey’s multiple comparison test. A test for linear trends in GraphPad Prism 5 was also used to analyze dose dependence. Pearson’s correlation coefficient and the linear regression among bDNA-RNA, bDNA-lysate, and RT-PCR results were analyzed with Excel (Microsoft, Tokyo, Japan). The differences between HA and HA-cell and the differences between HA and HA-v were determined statistically with an unpaired *t* test in GraphPad Prism 5. Significance is denoted with asterisks or daggers (*, † P < 0.05, **, †† P < 0.01, ***, ††† P<0.001).

## Results

### Conventional animal testing

**Fig 1:**
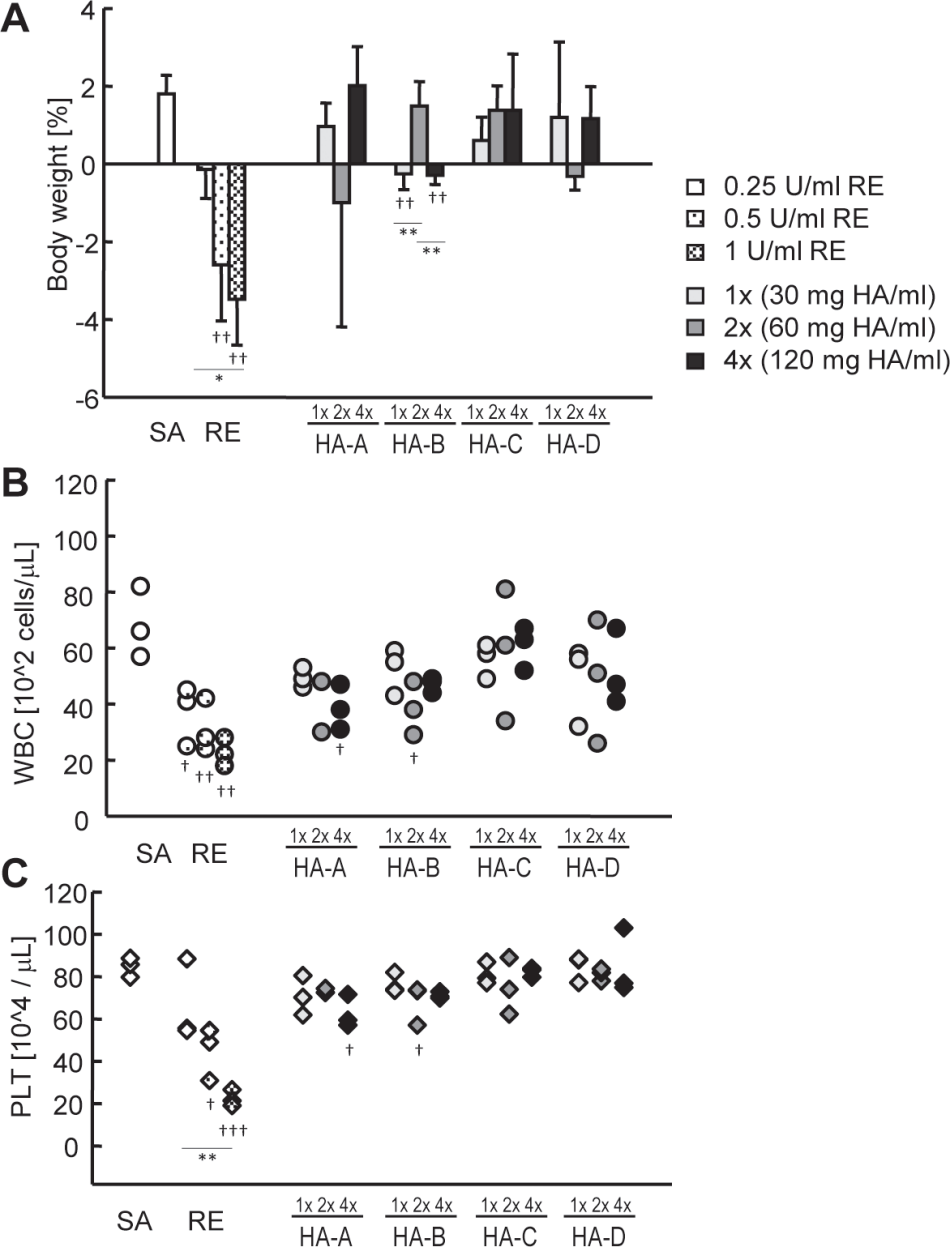
Abnormal toxicity test (ATT) and hematological tests of influenza vaccine-treated rats. The effects of RE, HA bulk materials, and SA treatment were measured using ATT, LTT and platelet (PLT) counts. Three rats per group were used, and were analyzed at day 1 after vaccine administration. (A) Changes in rat body weight were assessed as the percentage increase or decrease, and are indicated by the mean change ± S.D. WBC (B) and PLT (C) of peripheral blood were measured and plotted. Each symbol corresponds to an individual rat. Symbols filled with a few spots, a medium amount of spots, and many spots indicate 0.25, 0.5, and 1 U/ml of RE. Pale gray, gray, and black in each symbol indicate 1x, 2x, and 4x concentrated HA bulk materials. One blood sample of HA-A (2x) was clotted and omitted from LTT and PLT counts. * P < 0.05, ** P < 0.01. † P < 0.05 versus SA, †† P < 0.01 versus SA, ††† P < 0.001 versus SA.

To assess the sensitivity of animal safety tests and gene expression analysis, we prepared the national toxicity reference (RE) which was used as the reference for the leukopenic toxicity test (LTT), and was expected to show strong response. RE was made of inactivated influenza virus, and used as an alternative to a whole virion influenza vaccine. We also prepared HA bulk materials which were expected to show moderate or weak response. RE and HA bulk materials were serially diluted, and were subjected to the abnormal toxicity test (ATT). After samples were injected into the rat peritoneum, the weight was measured 1 day after injection. Compared with saline (SA), RE (0.5–1 U/ml) showed significant weight loss of treated rats (Fig 1A, spotted columns). The individual variability was relatively small, and dose dependency was clearly shown. For the LTT and platelet (PLT) counts, peripheral blood samples were collected from the same rats that underwent ATT. Compared with SA, RE (0.25–1 U/ml) showed a significant decrease in white blood cell (WBC) and PLT counts in a dose dependent matter (Fig 1B and 1C, spotted symbols).

The data from the HA bulk materials from four manufactures (HA-A to HA-D) showed clear contrast to the data from RE. Most of the HA bulk materials did not show significant weight loss as compared with SA, although HA-B (1x) and HA-B (4x) showed significant weight loss (Fig 1A). The weight loss of these sample-treated groups might be due to animal individual variability, and might not reflect the responsiveness of components containing in HA bulk materials, because the weight loss did not show dose-dependency. In WBC and PLT counts, significant decrease was observed in HA-A (4x) and HA-B (2x) treated groups (Fig 1B and 1C). These results suggested that animal safety tests could show dose-dependent response by RE, but could not show significant response by HA bulk materials.

### Gene expression profiles of vaccinated rats

**Fig 2:**
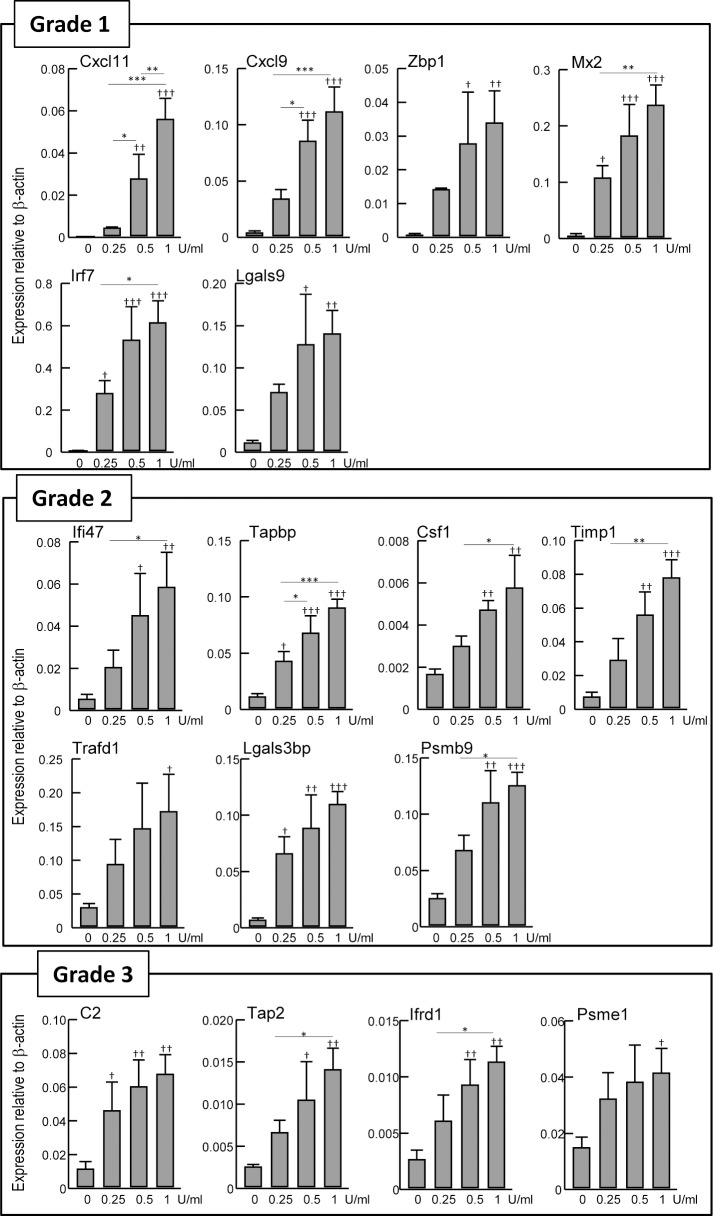
Gene expression analysis of RE treated rats. One lung lobe was obtained from rats treated with various concentrations of RE in Fig 1, and expression levels of genes were analyzed by bDNA-RNA, and were shown relative to β-actin. Three rats per group were used. Values are expressed as mean ± S.D. * P < 0.05, ** P < 0.01, *** P < 0.001. † P < 0.05 versus SA, †† P < 0.01 versus SA, ††† P < 0.001 versus SA.

**Fig 3:**
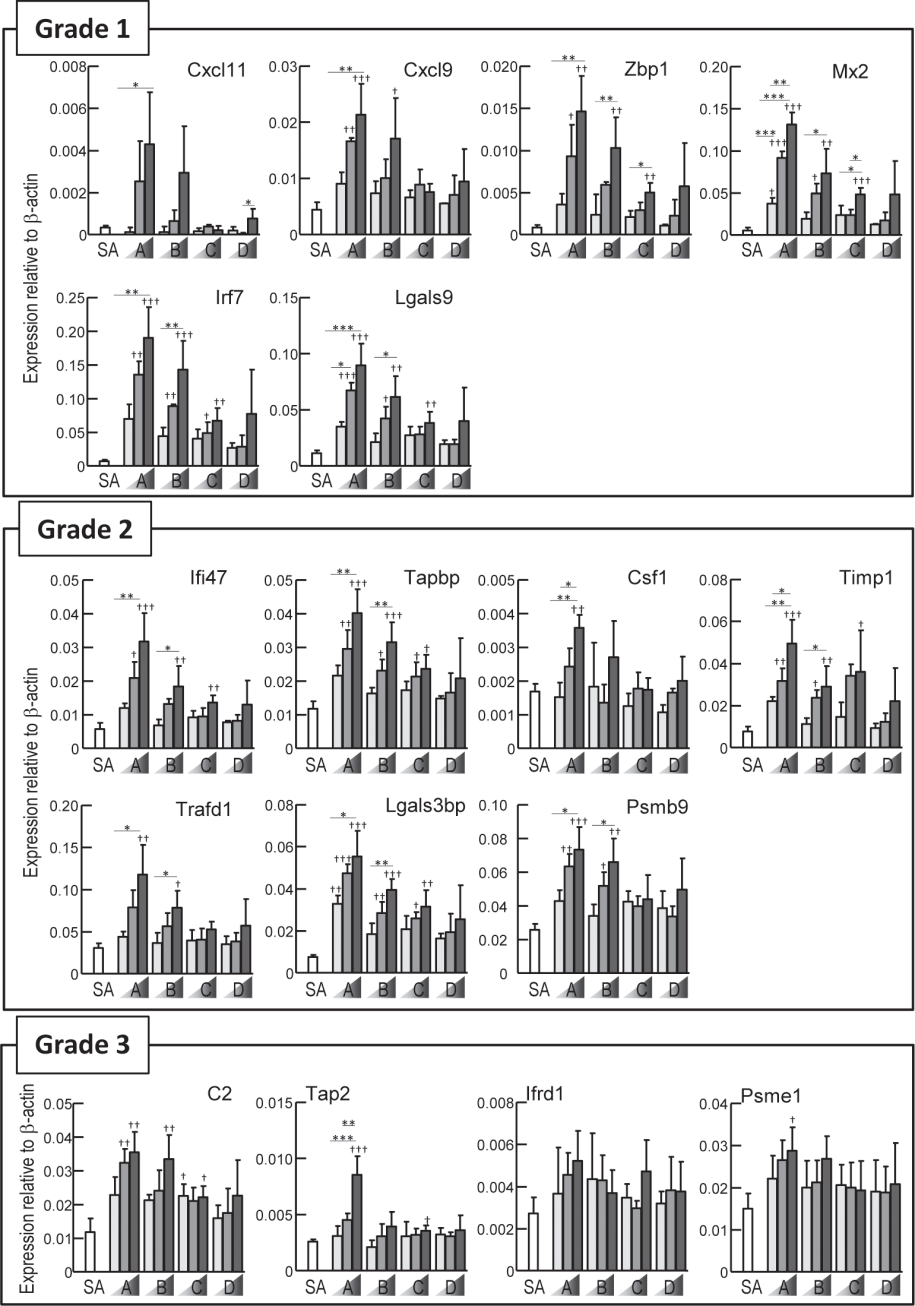
Gene expression analysis of HA bulk material treated rats. One lung lobe was obtained from rats treated with various concentrations of HA bulk material in Fig 1, and expression levels of genes were analyzed by bDNA-RNA, and were shown relative to β-actin. Same data was used in SA of Fig 3 and 0 U/ml RE of Fig 2. Three rats per group were used. Values are expressed as mean ± S.D. * P < 0.05, ** P < 0.01, *** P < 0.001. † P < 0.05 versus SA, †† P < 0.01 versus SA, ††† P < 0.001 versus SA.

Total RNA was prepared from the lungs of the vaccinated rats, and gene expression profiles were analyzed by a bDNA-based method (bDNA-RNA). In our previous works, we identified 17 genes as biomarkers for use in the quality control of influenza vaccines [20, 24]. They were provisionally classified into three classes according to the expression ratios between split and whole virion vaccines (Table 1) [23]. For the provisional classification of the biomarkers, the LTT criterion was applied: leukopenic toxicity levels of the split vaccine should be less than 20% of RE [17]. *Cxcl11*, *Cxcl9*, *Zbp1*, *Mx2*, *Irf7* and *Lgals9* were classified as “Grade 1”, because their relative expression ratios between the split and whole virion vaccines were less than 10%. Similarly, *Ifi47*, *Tapbp*, *Csf1*, *Timp1*, *Trafd1*, *Lgals3bp* and *Psmb9* were classified as “Grade 2”, because their relative expression ratios were less than 20%. *C2*, *Tap2*, *Ifrd1* and *Psme1* were classified as “Grade 3”, because their relative expression ratios were less than 40%. After RE treatment, the expression of all biomarker genes in Grade 1–3 was upregulated in a dose-dependent manner, correlating well with the ATT and LTT results (Fig 2). Fig 3 shows the gene expression results after rats were injected with concentrated HA bulk materials (HA-A through HA-D). Almost all genes were upregulated in HA-A and HA-B treated groups in a dose-dependent manner. *Ifrd1* (Grade 3) was not upregulated upon any HA bulk materials. In response to HA-C, the expression of *Zbp1*, *Mx2*, *Irf7*, and *Lgals9* (Grade 1), *Ifi47*, *Tapbp*, *Timp1*, and *Lgals3bp* (Grade 2), and *C2* (Grade 3) was upregulated. These results suggested that gene expression analysis may have higher sensitivity than ATT and LTT.

### Introduction of a bDNA-based method (bDNA-lysate)

**Fig 4:**
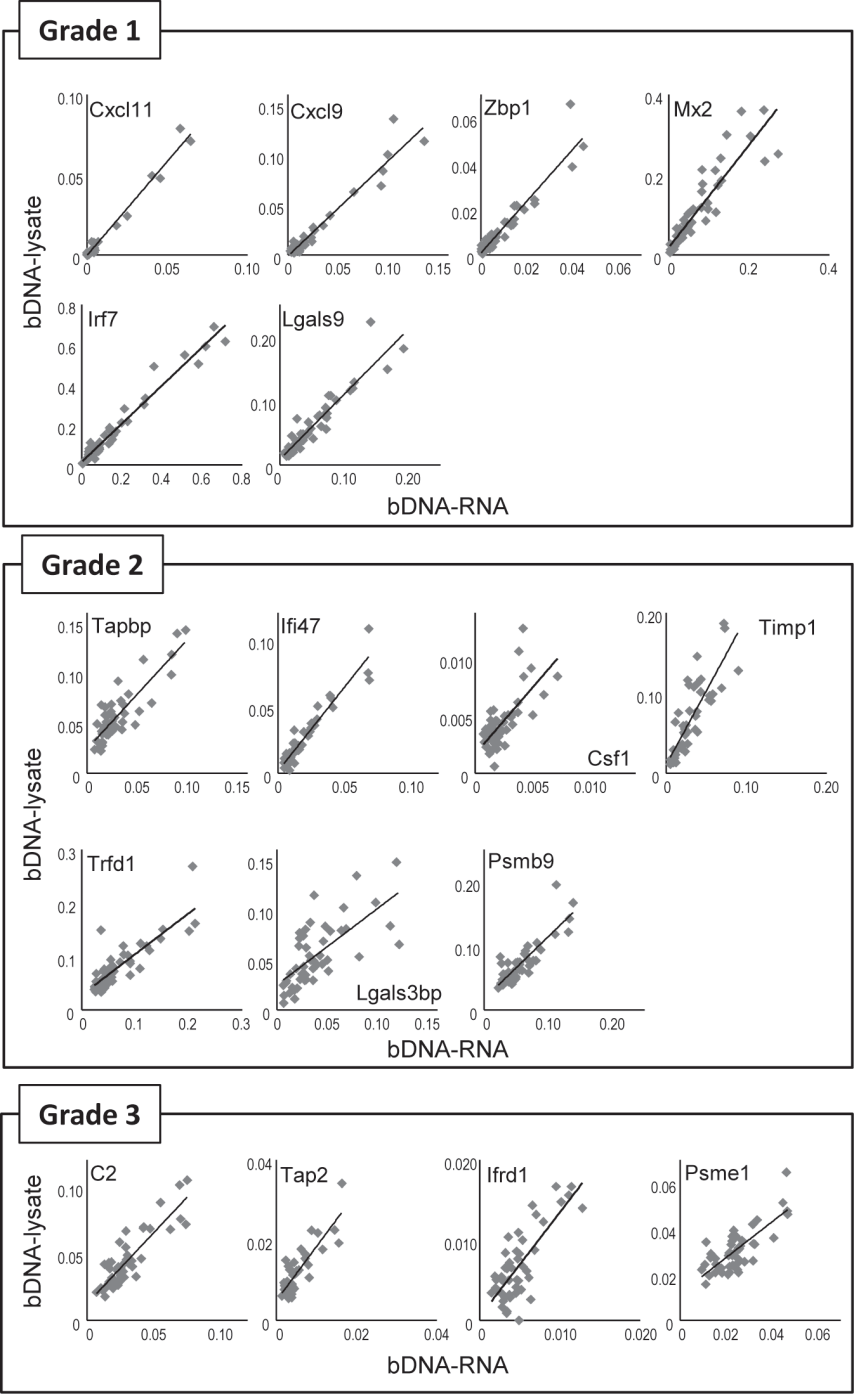
Validation analysis between bDNA-RNA and bDNA-lysate. Data of bDNA-RNA and bDNA-lysate were plotted against each other. The expression levels of genes were measured using purified RNA by bDNA-RNA, and using lysate by bDNA-lysate. Data of bDNA-RNA were from Figs 2 and 3. Expression levels were shown relative to β-actin.

To simplify the RNA extraction step, we next applied gene expression analysis directly to lung lysate (bDNA-lysate, ref.^21^). Lung lysate was prepared from the same rats as those in Figs 2 and 3, and subjected to gene expression analysis. The results for bDNA-RNA and bDNA-lysate correlated well (Fig 4), with Pearson’s correlation coefficient > 0.7 for 16 genes (Table 2). However, for 11 genes, the slopes of the linear regressions were not within the range of 0.9–1.1, suggesting that measured values were different (Table 2, see Discussion). The results for bDNA-lysate also correlated with the results for RT-PCR with Pearson’s correlation coefficient > 0.7 for 15 genes (Table 3). We also confirmed the correlation between the RT-PCR and bDNA-RNA results, in which Pearson’s correlation coefficient was > 0.7 for 15 genes (Table 4, ref. 23). These results suggest that bDNA-lysate could be applied for gene expression analysis of vaccine-treated rat lungs.

**Table 2:** bDNA-RNA versus bDNA-lyste.

Class	Grade 1						Grade 2							Grade 3			
gene	*Cxcl11*	*Cxcl9*	*Zbp1*	*Mx2*	*Irf7*	*Lgals9*	*Ifi47*	*Tapbp*	*Csf1*	*Timp1*	*Trafd1*	*Lgals3bp*	*Psmb9*	*C2*	*Tap2*	*Ifrd1*	*Psme1*
**Pearson's correlation coefficient (R)**	0.990	0.971	0.948	0.908	0.982	0.936	0.954	0.864	0.709	0.837	0.842	0.691	0.862	0.874	0.854	0.776	0.770
**slope of linear regression**	1.170	0.947	1.129	1.277	0.952	1.012	1.262	1.089	1.158	1.844	0.764	0.760	0.970	1.063	1.348	1.300	0.779
**y-intercept of linear regression**	0.000	-0.001	0.001	0.019	0.014	0.009	0.002	0.024	0.002	0.010	0.028	0.025	0.017	0.013	0.005	0.001	0.012

**Table 3:** bDNA-lysate versus RT-PCR.

Class	Grade 1						Grade 2							Grade 3			
gene	*Cxcl11*	*Cxcl9*	*Zbp1*	*Mx2*	*Irf7*	*Lgals9*	*Ifi47*	*Tapbp*	*Csf1*	*Timp1*	*Trafd1*	*Lgals3bp*	*Psmb9*	*C2*	*Tap2*	*Ifrd1*	*Psme1*
**Pearson's correlation coefficient (R)**	0.960	0.942	0.939	0.921	0.981	0.958	0.973	0.859	0.583	0.814	0.770	0.690	0.883	0.863	0.719	0.823	0.776
**slope of linear regression**	1.035	0.313	1.549	0.497	0.203	2.151	0.580	0.204	0.394	0.585	2.416	0.054	0.395	0.346	0.361	0.386	0.073
**y-intercept of linear regression**	-0.001	-0.001	0.000	0.022	0.024	0.007	0.005	0.025	0.000	0.013	0.011	0.030	0.028	0.001	0.005	0.000	0.015

**Table 4:** bDNA-RNA versus RT-PCR.

Class	Grade 1						Grade 2							Grade 3			
gene	*Cxcl11*	*Cxcl9*	*Zbp1*	*Mx2*	*Irf7*	*Lgals9*	*Ifi47*	*Tapbp*	*Csf1*	*Timp1*	*Trafd1*	*Lgals3bp*	*Psmb9*	*C2*	*Tap2*	*Ifrd1*	*Psme1*
**Pearson's correlation coefficient (R)**	0.971	0.965	0.924	0.932	0.968	0.933	0.965	0.952	0.449	0.895	0.828	0.903	0.863	0.888	0.828	0.790	0.676
**slope of linear regression**	0.886	0.329	1.281	0.358	0.207	1.939	0.435	0.179	0.186	0.292	2.863	0.064	0.343	0.293	0.263	0.221	0.063
**y-intercept of linear regression**	0.000	0.000	0.000	0.008	0.014	0.003	0.004	0.002	0.000	0.004	-0.014	0.010	0.018	-0.006	0.000	0.001	0.010

### Quality control of next generation vaccines

**Fig 5:**
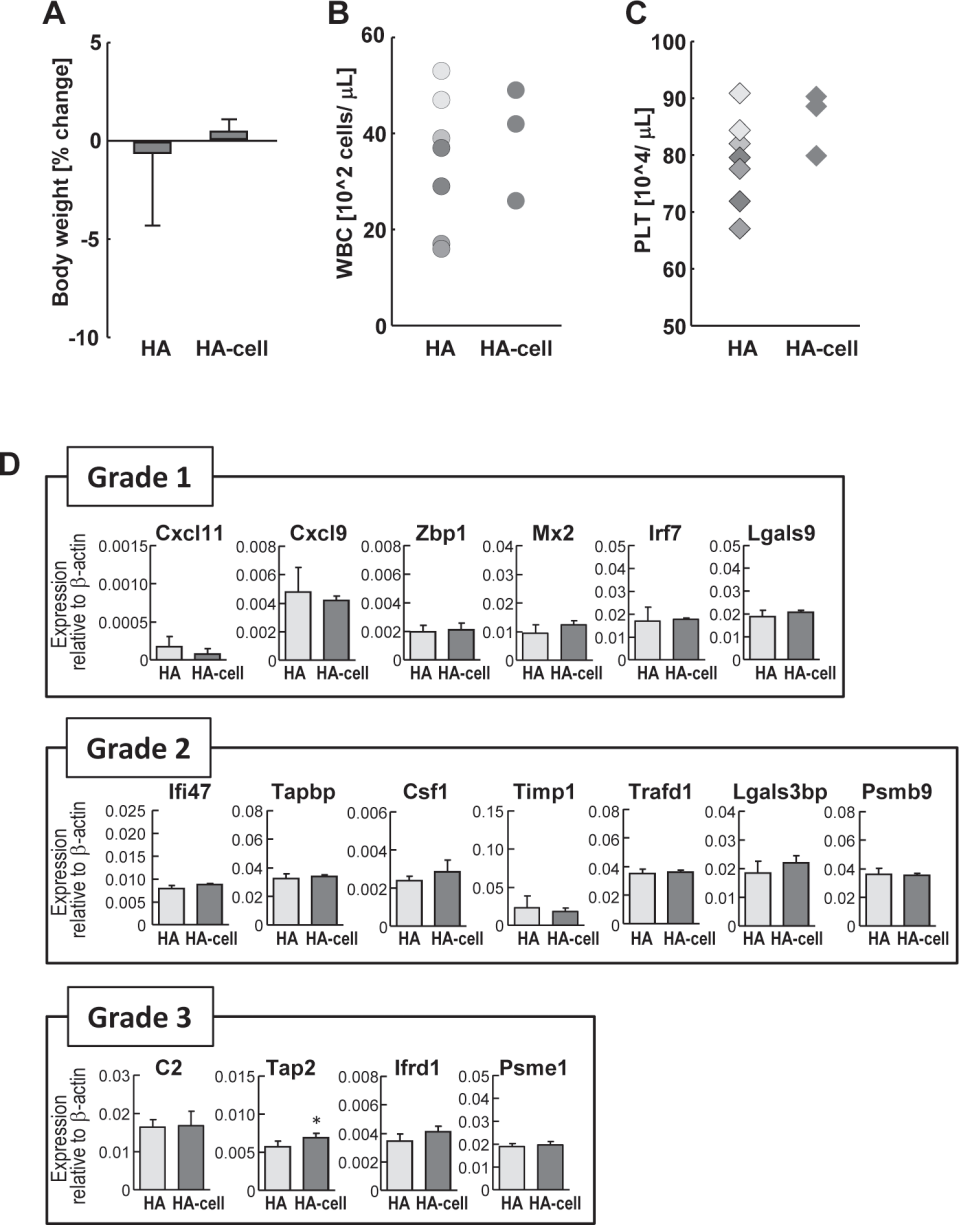
Animal test and gene expression analysis of HA-cell vaccine treated rats. The effects of HA-cell were measured using ATT, LTT, PLT counts, and gene expression analysis. All rats were analyzed at day 1 after vaccine injection. Eight rats were used for HA, and three rats were used for HA-cell. (A) Changes in rat body weight were assessed as the percentage increase or decrease, and are indicated by the mean change ± S.D. (B, C) WBC and PLT of peripheral blood were measured and plotted in graphs. Each symbol corresponds to an individual rat. Difference in symbol colors represents different batches of HA. One blood sample of HA was clotted and omitted from LTT and PLT counts. (D) The expression levels of genes were analyzed by bDNA-lysate, and were shown relative to β-actin. Values are expressed as mean ± S.D. * P < 0.05.

**Fig 6:**
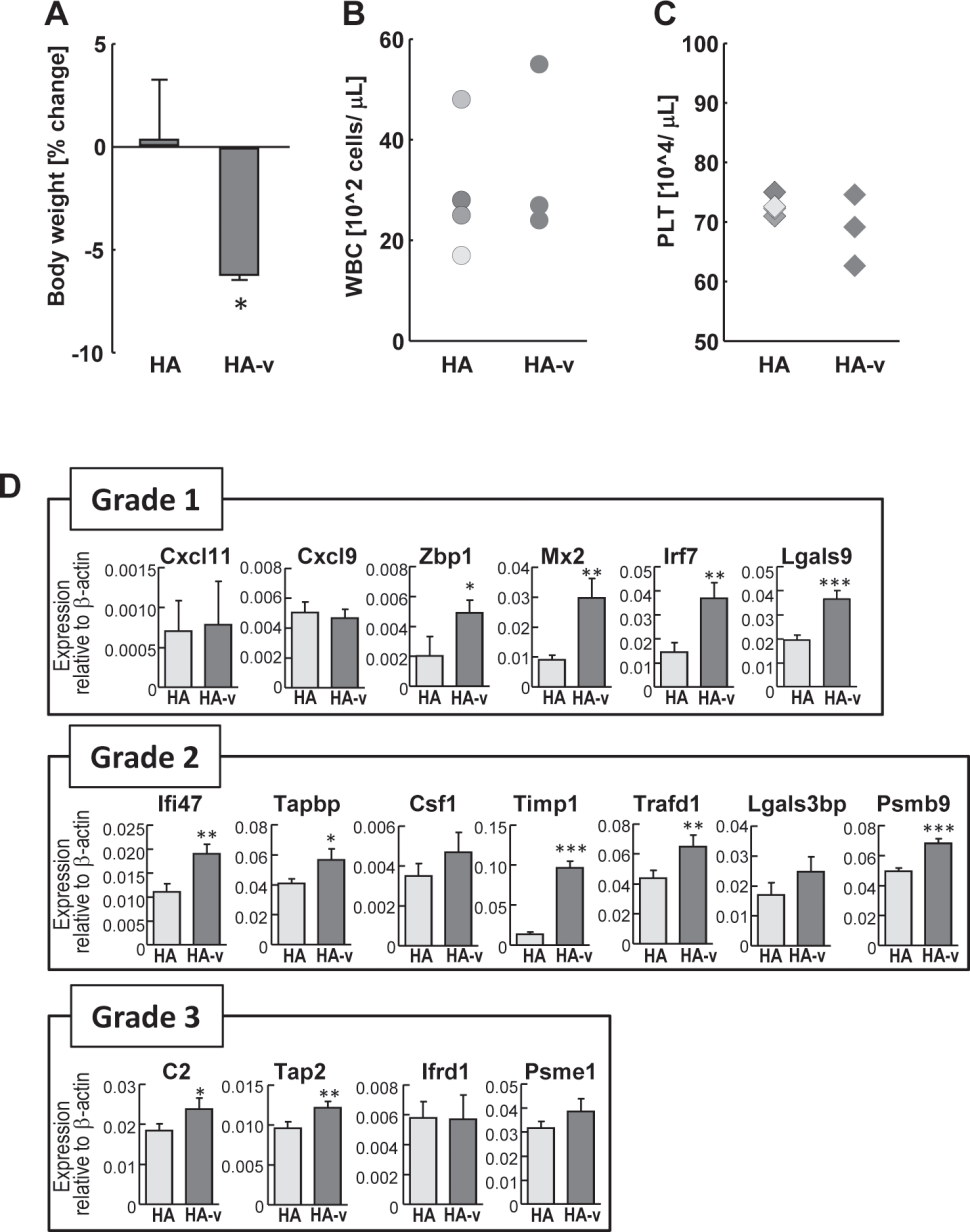
Animal test and gene expression analysis of HA-v vaccine treated rats. The effects of HA-v were measured using ATT, LTT, PLT counts, and gene expression analysis. All rats were analyzed at day 1 after vaccine injection. Four rats were used for HA, and three rats were used for HA-v. (A) Changes in rat body weight were assessed as the percentage increase or decrease, and are indicated by the mean change ± S.D. (B, C) WBC and PLT of peripheral blood were measured and plotted in graphs. Each symbol corresponds to an individual rat. Difference in symbol colors represents different batches of HA. (D) The expression levels of genes were analyzed by bDNA-lysate, and were shown relative to β-actin. Values are expressed as mean ± S.D. * P < 0.05, ** P < 0.01, *** P < 0.001.

Although many seasonal influenza vaccines are split-type, influenza vaccines of the next generation have been recently authorized and introduced into market. To demonstrate whether the biological effect of next generation vaccines were similar to the current split vaccines, and whether expression profiles of our biomarkers were correlated to the biological responsiveness, animal testing (ATT and LTT) were perform to be compared by gene expression analysis. Two types of next generation vaccines were used for analysis: HA vaccine manufactured from a continuous Vero cell line (HA-cell), and virosomal-adjuvanted HA vaccine (HA-v). Four batches of split vaccines manufactured from fertilized eggs (HA) were used as control; each batch was from distinct manufactures. As shown in Fig 5A, HA-cell did not show any weight loss compared with HA in the ATT assay. WBC and PLT counts of HA-cell injected samples were within the range of the HA sample, and no significant differences were observed between HA and HA-cell (Fig 5B and 5C). Gene expression pattern correlated well the ATT and LTT and no significant differences were shown between HA and HA-cell, except for *Tap2* which was slightly upregulated compared with the HA-treated group (Fig 5D). In contrast, HA-v showed significant weight loss in ATT (Fig 6A). However, no significant differences of WBC and PCT counts were observed between HA and HA-v (Fig 6B and 6C). For gene expression analysis, 11 genes, *Zbp1*, *Mx2*, *Irf7*, and *Lgals9* (Grade 1), *Ifi47*, *Tapbp*, *Timp1*, *Trafd1*, and *Psmb9* (Grade 2), *C2* and *Tap2* (Grade 3), were significantly upregulated in the HA-v treated group compared with the HA-treated group (Fig 6D, asterisks), which is consistent with the results of ATT. These data suggest that gene expression analysis can reflect the results of animal testing, and could be introduced for the quality control of influenza vaccines of the next generation including HA-cell and HA-v.

## Discussion

Quality control of influenza vaccines has been performed using animal tests, such as ATT and LTT. However, conventional animal tests require a relatively long period of time and large numbers of animals; seven days in ATT and ten mice per group in LTT. In addition, the low reproducibility that usually occurs in animal experiments is problematic. Thus, the development of a new quality control test is desired. In this study, we showed that the expression patterns of 17 biomarkers had high sensitivity for assessing the condition of influenza vaccine treated rats. Further, the 17 biomarkers could be analyzed by a bDNA-lysate method that is more straightforward and quicker than traditional RNA expression profiling, because no RNA purification or amplification steps are needed, and target genes can be analyzed simultaneously. A bDNA-based assay has been recently used for gene expression analysis [25–28], and we expect that concurrent expression analysis of biomarkers such as a bDNA-based assay will shorten the quality control test period of influenza vaccines in the future.

As shown in Fig 4 and Tables 2–4, data from bDNA-RNA, bDNA-lysate, and RT-PCR were well correlated with each other. However, the slopes of the linear regressions of some genes were not within the range of 0.9–1.1, indicating difference in the results of the assays of some genes. The differences in the values measured with bDNA-RNA and bDNA-lysate may be attributable to the extraction efficiency of the target RNA. The differences in the values measured with RT-PCR and the bDNA-based analysis are attributable to the effective measurement range of the bDNA-based assay: because the expression levels of all genes need to be measured simultaneously within the effective data range of luminometer in bDNA-based assay, the assay had a lower sensitivity to genes with high expression and also had a higher sensitivity to genes with low expression. This explains the difference in measured values between the RT-PCR and bDNA-based assays. The results of Csf1 showed low correlation between RT-PCR and bDNA-based analyses (R = 0.583 for bDNA-lysate versus RT-PCR, and R = 0.449 for bDNA-RNA versus RT-PCR). This may be because Csf1 showed a relatively small expression change upon sample treatment, and also because of the individual variability of rats. Although some aspects should be improved, the overall expression pattern of 17 genes was quite reproducible among the different assays.

It is desirable for the criteria used to evaluate gene expression analyses are equivalent to the criteria used to evaluate the currently used tests. In the ATT, no statistically significant difference (p = 0.01) in weight loss must be observed between the test animals and the parental group. In the LTT, the leukopenic toxicity levels must be less than 20% of RE. However, it is difficult to determine the thresholds that can be used as corresponding criteria in the gene expression analysis. It might be helpful to establish a parental group for comparison, in which the rats are treated with previously satisfactory batches of the same kind of vaccine. A statistical comparison of the gene expression profiles of the animals treated with the test sample and the parental group could be used to evaluate every batch of vaccine and to check the batch-to-batch consistency. Further research is still required to set the thresholds of gene expression for vaccine acceptability.

In previous studies, we provisionally classified our biomarkers into three classes according to the inducible expression levels [23]. The expression of *Cxcl11* and *Cxcl9* (Grade 1), *Csf1* and *Lgals3bp* (Grade 2), *Ifrd1* and *Psme1* (Grade 3) were not induced by HA-cell and HA-v. These data suggested that *Cxcl11*, *Cxcl9*, *Csf1*, *Lgals3bp*, *Ifrd1* and *Psme1* might be useful biomarkers to check the clear separation of HA vaccine from for whole virion influenza particle.

Interestingly, we identified *Irf7* and *Mx2* (Grade 1) as biomarkers not only for influenza vaccine, but also for pertussis vaccine [24]. *Irf7* and *Mx2* are considered ISGs. After recognition of virus components by pattern recognition receptors, transcription factors including Irf3 become activated, translocate into the nuclei, and activate the expression of interferons and ISGs. Irf7 is a close relative of Irf3 and acts to further expression of interferons while Irf3 is constitutively expressed. Stable knock-down of *Irf7* in MDCK cells had been reported to enhance the production of influenza viruses [29]. *Mx2* and its homolog *Mx1* possess antiviral activity [30] and have shown to be upregulated after influenza virus infection in cotton rat’s lung [31]. Interferons and ISGs are also induced by most bacterial pathogens [32], although the antibacterial functions have not been fully understood. It is noteworthy that *Irf7* and *Mx2* are upregulated upon whole virion influenza vaccine which showed leukopenia in treated animals, and are downregulated upon whole cell pertussis vaccine which showed leukocytosis in treated animals [19–21]. Furthermore, Nakaya et al have reported the upregulation of *Irf7* and *Mx2* after *in vitro* stimulation of human peripheral blood cells with live attenuated influenza vaccine, and also, after stimulation with live attenuated yellow fever vaccine [33]. It may be useful if *Irf7* and *Mx2* could be applied for a variety of influenza vaccines, and further, could be applied for vaccines for other viruses and bacteria.

Aluminum-based adjuvants have been widely used for seasonal vaccines. Novel adjuvants are relatively well-studied, and some are in practical use for pandemic vaccines [34–36]. Virus-like particle- and nanoparticle-based vaccines are also exciting technologies for effective vaccination for seasonal and pandemic vaccines [37, 38]. Other vaccine formulations will follow in the future, and the importance of quality control of adjuvant-containing vaccines is increasing. We have previously suggested that *Cxcl9*, *Trafd1*, and *C2* could be candidate genes for the evaluation of aluminum adjuvant-containing vaccines [23]. In this study, we have reported that *Zbp1*, *Mx2*, *Irf7*, *Lgals9* (Grade 1), *Ifi47*, *Tapbp*, *Timp1*, *Trafd1*, *Psmb9* (Grade 2), *C2* and *Tap2* (Grade 3) could be candidate markers for assessing the difference between HA and HA-v. In the future, we hope to investigate whether the set of 17 biomarkers could be applicable for the quality control of influenza vaccines containing various adjuvants.
